# Machine Learning–Based Time in Patterns for Blood Glucose Fluctuation Pattern Recognition in Type 1 Diabetes Management: Development and Validation Study

**DOI:** 10.2196/45450

**Published:** 2023-05-26

**Authors:** Nicholas Berin Chan, Weizi Li, Theingi Aung, Eghosa Bazuaye, Rosa M Montero

**Affiliations:** 1 Informatics Research Centre Henley Business School University of Reading Reading United Kingdom; 2 Royal Berkshire NHS Foundation Trust Reading United Kingdom; 3 King's College London London United Kingdom

**Keywords:** diabetes mellitus, continuous glucose monitoring, glycemic variability, glucose fluctuation pattern, temporal clustering, scalable metrics

## Abstract

**Background:**

Continuous glucose monitoring (CGM) for diabetes combines noninvasive glucose biosensors, continuous monitoring, cloud computing, and analytics to connect and simulate a hospital setting in a person’s home. CGM systems inspired analytics methods to measure glycemic variability (GV), but existing GV analytics methods disregard glucose trends and patterns; hence, they fail to capture entire temporal patterns and do not provide granular insights about glucose fluctuations.

**Objective:**

This study aimed to propose a machine learning–based framework for blood glucose fluctuation pattern recognition, which enables a more comprehensive representation of GV profiles that could present detailed fluctuation information, be easily understood by clinicians, and provide insights about patient groups based on time in blood fluctuation patterns.

**Methods:**

Overall, 1.5 million measurements from 126 patients in the United Kingdom with type 1 diabetes mellitus (T1DM) were collected, and prevalent blood fluctuation patterns were extracted using dynamic time warping. The patterns were further validated in 225 patients in the United States with T1DM. Hierarchical clustering was then applied on time in patterns to form 4 clusters of patients. Patient groups were compared using statistical analysis.

**Results:**

In total, 6 patterns depicting distinctive glucose levels and trends were identified and validated, based on which 4 GV profiles of patients with T1DM were found. They were significantly different in terms of glycemic statuses such as diabetes duration (*P*=.04), glycated hemoglobin level (*P*<.001), and time in range (*P*<.001) and thus had different management needs.

**Conclusions:**

The proposed method can analytically extract existing blood fluctuation patterns from CGM data. Thus, time in patterns can capture a rich view of patients’ GV profile. Its conceptual resemblance with time in range, along with rich blood fluctuation details, makes it more scalable, accessible, and informative to clinicians.

## Introduction

### Background

Diabetes mellitus (DM) is a lifelong condition owing to elevated glucose concentration in blood and has long been a major global public health issue. According to the International Diabetes Federation, the number of people with diabetes has risen from 151 million in 2000 to 537 million in 2021 and is projected to reach 783 million by 2045 [1]. The World Health Organization estimated that 1.5 million deaths were directly caused by diabetes in 2019, making it the ninth leading cause of death [2]. Before the introduction of smart and connected health and hence continuous glucose monitoring (CGM) wearable devices, self-monitoring of blood glucose (BG) level played a crucial role in the management of patients with DM. However, a landmark paper in 2008 revealed that patients rarely measured glucose levels after meals or overnight, which led to postprandial hyperglycemia within the group of patients [3]. Results from a multicenter randomized control trial further illustrated that the use of CGM is associated with improved glycemic control in adults with type 1 DM (T1DM). CGM for diabetes combines noninvasive glucose biosensors, continuous monitoring, cloud computing, and analytics to connect and simulate a hospital setting in a person’s home. It uses sensors to measure glucose levels just beneath the surface of the skin and sends data wirelessly to the users’ compatible smart device or receiver [4]. CGM works as a connected and closed-loop system that enables patients to modify their insulin dosages based on their glucose trends in a timely manner. With the advancement of technology, CGM has become much more accurate and assessable, making it a vital tool for patients with DM to manage their BG level. According to a survey in 2019, the percentage of CGM users with T1DM in the US T1D Exchange registry has increased from 7% in 2010 to 30% in 2018 [5]. A systematic review and meta-analysis in 2019 concluded that the use of CGM over self-monitoring is beneficial in terms of several clinical outcomes [6].

### Average BG to Glycemic Variability

As suggested by Huisman et al [7] and characterized by Bookchin and Gallop [8], glycated hemoglobin (HbA_1c_) level has been the gold standard for testing BG intensity and defining diabetes since its proposal. It is a measure of average glucose within a person over the previous 8 to 12 weeks [9] and has been adopted by major clinical guidelines for managing the glycemic status of patients with T1DM and diagnosing and screening people who are at risk of type 2 DM [10-12].

The introduction of CGM opened up new areas of research for BG control owing to the sheer volume of BG data it collects. Despite the well-recognized evidence and wide use of HbA_1c_ level, there has been increasing research interest in glycemic variability (GV), which is based on CGM data, arguing that GV contains additional diagnostic and prognostic value that could not be fully captured by HbA_1c_ measurement. BG variability, also known as GV, refers to the degree of oscillation in BG levels [13]. Patients with diabetes often rely heavily on continuous medication intake to maintain BG at a normal and stable level. However, this is often difficult as food consumption would lead to a spike in BG, whereas the use of excessively intensive medication could lead to hypoglycemia. As HbA_1c_ measurement fails to effectively capture these oscillations, HbA_1c_ level alone is not an ideal indicator of an individual patient’s glycemic control [14]. Studies have been conducted to evaluate the diagnostic and prognostic value of GV. It is shown that high GV is associated with high risk of microvascular and macrovascular complications [15,16], high mortality in patients who are critically ill [17-19], and high incidence of neurological outcomes [20]. A systematic review and meta-analysis conducted by Gorst et al [21] indicated that high GV is associated with increased risk of renal disease, cardiovascular events, retinopathy, ulceration, and mortality.

### Quantifying GV

Several methods have been proposed to capture GV from CGM data. SD and coefficient of variation (COV) are the 2 most prevalent metrics in the field owing to their ease of calculation and relative understandability. However, they are often criticized as a statistically biased metric to represent GV because BG readings do not follow a normal distribution and tend to skew toward hyperglycemia, especially in patients with diabetes [22,23]. In addition, they do not incorporate the information about time and sequences of readings in their calculations. As such, even if one randomly reorders a set of BG readings to obtain drastically different glycemic curves, the SD and COV would still remain the same.

Time in range (TIR) has been proposed by existing studies as a way to indirectly capture GV [24-29]. TIR refers to the daily proportion of time one’s glucose level falls within given target ranges with breakpoints typically at 3, 3.9, 10, and 13.9 mmol/L [29]. The major strengths of TIR are that it can be readily computed and it is much more intuitive to clinicians, while still, to some extent, able to capture how much a person’s BG deviates from the target range. So far, studies have shown that TIR alone is associated with a wide range of outcomes, such as diabetic retinopathy [26] and various neonatal outcomes [30]. A conference conducted in 2018 reached a consensus that outlined the use of CGM and related glycemic metrics to improve glucose management [27,28]. Despite the widely recognized strengths of TIR, its aggregated nature inevitably implies that temporal fluctuation information from CGM data is left unused, which was shown to contain further prognostic value. In particular, as TIR also disregards the order in which the glucose measurements were made, it fails to provide details about specific glycemic patterns that occurred in one’s CGM history.

Most metrics fail to account for the sequences of BG measurements without the use of sophisticated statistical or machine learning models because that would involve recognizing a trend or pattern within a time series of BG data. Thus, machine learning models have also been proposed to compute GV. Struble [31] and Marling et al [32] applied support vector regression to model the data points from CGM and computed GV based on the difference between actual and modeled data points. Eljil et al [33] suggested the use of time-sensitive artificial neural networks to predict hypoglycemic events, whereas Mani et al [34] used random forest models to predict the risk of type 2 DM. Furthermore, Hall et al [35] defined 3 glucose fluctuation patterns, namely low, medium, and high variability, by using dynamic time warping (DTW). A list of analytic methods and metrics for quantifying GV in existing literature is summarized in Table 1. Although these machine learning–based methods successfully used the temporal information embedded in CGM data, they were criticized to be “not well understood in clinical practice” [36], which remains as a major hurdle that hinders clinicians from applying these methods in practice.

**Table 1:** Summary of metrics and analytics methods for assessing glycemic variability (GV).

Metrics and analytic methods	Related publications	Strengths	Limitations
SD	Krinsley [19]	Simplicity	Tend to be skewed and does not adjust for mean BG^a^ level Does not account for temporal information Limited capability of interpreting GV profiles with only a single value
COV^b^	Rodbard [37] and Rama Chandran et al [38]	Simplicity and adjusts for mean	Does not account for temporal information Limited capability of interpreting GV profiles with only a single value
TIR^c^	Omar et al [24], Beck et al [25], Lu et al [26], Beyond A1C Writing Group [27], Battelino et al [28], and Advani [29]	Simplicity	Does not account for sequence of BG measurements
IQR	McDonnel et al [39]	Simplicity	Does not adjust for mean BG level Does not account for temporal information Limited capability of interpreting GV profiles with only a single value
Range	Oh et al [20]	Simplicity	Tend to be skewed and does not adjust for mean BG level Does not account for temporal information Limited capability of interpreting GV profiles with only a single value
MAGE^d^	Service [22] and Service et al [40]	Takes BG fluctuation owing to meal into account	Day based Does not adjust for mean BG level Does not account for sequences of BG measurements Limited capability of interpreting GV profiles with only a single value
LBGI^e^ and HBGI^f^	Kovatchev et al [41] and Hill et al [42]	Adjusts for BG skewness and measuring frequency	Does not account for sequences of BG measurements Ambiguities in BG variability level Limited capability of interpreting GV profiles with only a single value
SVR^g^	Struble [31] and Marling et al [32]	Accounts for temporal information	Limited capability of interpreting GV profiles with only 3 discrete levels Subject to clinicians’ experience in determining the variability levels; thus, lack of evidence
TS-ANN^h^	Eljil et al [33]	Accounts for temporal information	Limited capability of interpreting GV profiles with only a single value
RF^i^	Mani et al [34]	Accounts for temporal information	Limited capability of interpreting GV profiles with only a single value
Glucotypes	Hall et al [35]	Accounts for temporal information	Limited capability of interpreting GV profiles with only 3 discrete levels

^a^BG: blood glucose.

^b^COV: coefficient of variation.

^c^TIR: time in range.

^d^MAGE: mean amplitude of glycemic excursions.

^e^LBGI: low blood glucose index.

^f^HBGI: high blood glucose index.

^g^SVR: support vector regression.

^h^TS-ANN: time-sensitive artificial neural network.

^i^RF: random forest.

Furthermore, there has been scalability issues in existing CGM-related machine learning studies owing to the missingness of key variables in real-world application. For example, in most studies, participants are asked to manually log daily events (such as meal, stress level, exercise, and illnesses) and wear a wristband for collecting physiological data, which can potentially provide insights about GV management [43]. However, in real-world health care, most of the time, only routinely collected CGM and electronic patient record (EPR) data would be available for clinicians to make decisions about therapeutic pathways. A more scalable analytical framework is warranted to make full use of CGM data and capture detailed GV pattern to inform personalized therapeutic pathways. Computationally simple methods such as COV and TIR tend to show a narrow presentation of a patient’s GV profile but are more recognized among clinicians and used in more clinical studies. In contrast, despite being able to capture more information from CGM data, complex machine learning–based methods tend to be less intuitive for clinicians to apply in practice. Moreover, existing methods often express GV profile as a single value or a few discrete levels (usually high, medium, or low) and do not reveal detailed insights about any GV patterns that exist in the data.

In this study, we sought to address the scalability issues of machine learning–based GV management and fill the gap between the intuitiveness of simplistic methods, such as TIR, and comprehensiveness of machine learning methods to understand the underlying GV patterns in patients with T1DM who have been using wearable CGM. The aim of this paper was 2-fold. First, we sought to develop a novel and scalable analytics framework for efficient GV pattern recognition and attribution that provides a more comprehensive, easy-to-understand representation of a patient’s BG fluctuation profile, which cannot be solely captured by clinically established metrics such as HbA_1c_ level and TIR. Second, we sought to propose the use of time in patterns to depict GV profiles and show that it reveals additional insights about CGM data and patient characteristics. In the long run, we hope that having a rich and accessible representation of GV profile could serve as a step toward explainable artificial intelligence and the development of personalized therapeutic pathways for patients with T1DM.

## Methods

### Overview

**Figure 1:**
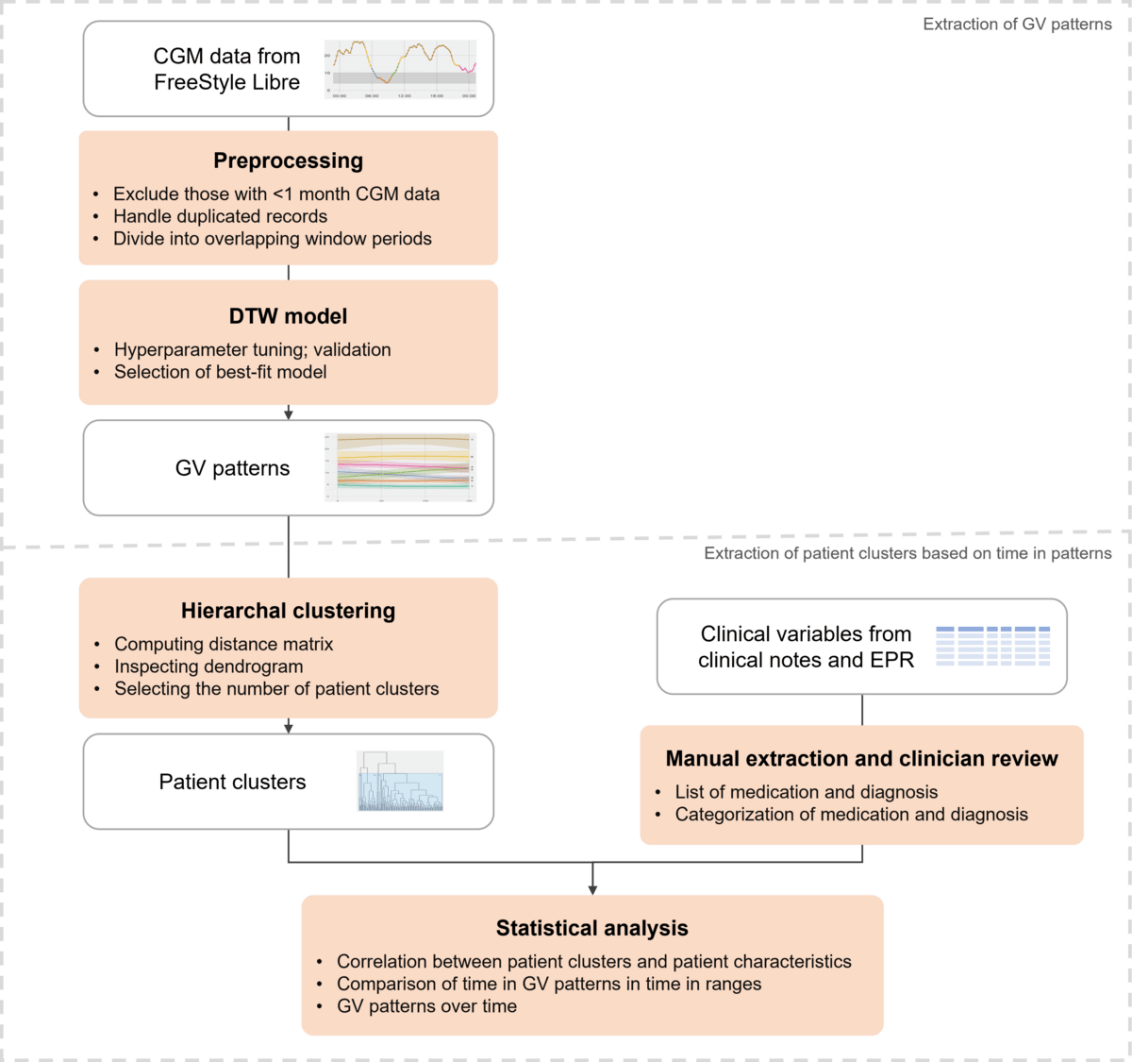
Analytical framework for glycemic variability (GV) pattern extraction and patient clustering from continuous glucose monitoring (CGM) data. DTW: dynamic time warping; EPR: electronic patient record.

The analysis of this study entailed two major parts (Figure 1): (1) extracting GV patterns from CGM and (2) clustering patients based on time in GV patterns and evaluating the clusters. For the first part, we gathered and filtered patients, extracted and cleaned their CGM data from monitoring devices, and then applied a machine learning algorithm called DTW. This enabled us to classify the given CGM data within a time window into one of the extracted patterns. In addition, we applied our methods to another CGM data set to externally validate our pattern extraction methods. In the second part, we computed the time spent in each pattern per patient. Clinical variables were gathered from EPR and clinical notes. Clustering methods were further applied on time in patterns to demonstrate its possible use cases by comparing the differences in clinical variables across clusters of patients. Finally, we evaluated the relationship between time in patterns developed using our method and well-established glycemic metrics.

All analyses were performed using R (version 4.0.3; R Core Team and the R Foundation for Statistical Computing), and the R package *dtwclust* (version 5.5.12, Sarda-Espinosa) was used for DTW-related analyses [44,45].

### Inclusion and Exclusion of Patients

All patients in this study attended the Centre for Diabetes and Endocrinology of a large hospital in the United Kingdom. The inclusion criteria included patients who (1) were diagnosed with T1DM and (2) were given a CGM device named FreeStyle Libre (FSL) before August 5, 2019, and had been using it for at least one month. Patients aged <18 years or patients with unavailable or missing National Health Service (NHS) identifiers were excluded from this study. Of 130 patients with available CGM data in FSL, 126 (96.9%) patients were included in this study.

### Collection of CGM Data

FSL flash glucose monitoring system was used to measure the interstitial fluid glucose level of included patients. It has been verified by the National Institute for Health and Care Excellence based on evidence from randomized controlled trials [46]. Patients were instructed by clinicians to use the device in accordance with the flash glucose monitoring guidelines suggested by NHS. When using FSL, patients continued to take insulin according to their insulin regimes and type of insulin they use. In addition, patients were arranged to have follow-up consultations every 3 to 6 months, depending on their clinical needs. Pragmatically, the glucose level was primarily measured and recorded once every 15 minutes.

Apart from the FSL data set, CGM data from the REPLACE-BG trial were used for external validation. The REPLACE-BG study is a multicenter randomized trial to evaluate the stand-alone effectiveness of CGM without confirmatory BG measurements in 225 adults with well-controlled T1DM [47]. The trial was chosen for external validation because it represented a patient group that is similar and relevant to this study in 3 ways. First, the REPLACE-BG cohort and our patient cohort both contained patients with T1DM who were using CGM and undergoing similar insulin treatment, which is an important inclusion criterion in this study. Second, the REPLACE-BG trial was conducted in the United States, whereas this study was conducted in the United Kingdom. The capability of our proposed methods to be applied to patients with different demographics can be tested. Third, as the REPLACE-BG trial included more patients and CGM measurements, it enabled us to validate our methods using a large sample size to demonstrate scalability.

### Retrieval and Preprocessing of Clinical Information From Clinical Notes and EPR

The FSL CGM data set did not contain clinical variables that are crucial to this analysis. Thus, clinical notes and EPR were used as sources of clinical information by mapping the participants’ NHS identifiers. All available clinical notes between August 5, 2009, and August 5, 2019, were manually reviewed, and the list of medication and diagnosis was extracted for each patient. Then, the list of medication and diagnosis was reviewed by clinicians at the Centre for Diabetes and Endocrinology to categorize them for further analysis (Tables S1 and S2 in Multimedia Appendix 1). In contrast, the latest laboratory test results, including HbA_1c_ level and estimated glomerular filtration rate, were retrieved from the EPR.

### GV Pattern Extraction With DTW

DTW was proposed by Berndt et al [48], and it aims to find patterns in time-series data. The DTW model takes several time-series data as input and outputs the time-series patterns extracted and the type of pattern to which each series belongs. The major strengths of DTW included its ability to handle unevenly spaced time-series data, which is prevalent in CGM data. Several researchers have applied DTW to discover clinical insights such as the prognostic value in CGM data [35], electrocardiograms [49], and genomic signals [50].

A few preprocessing steps were performed to transform the FSL CGM data into inputs for the DTW model. First, if multiple records were found within the same minute in the CGM data, the median value was considered. Second, we divided the CGM data of each patient into overlapping window periods. Any window periods that had <4 measurements per hour on average were discarded to improve model results. Third, hyperparameters of the DTW model, specifically, the duration of each window period and the percentage of overlap between consecutive windows, were tuned. A grid search was performed from a validation set over the 2 hyperparameters to determine the best combination that optimizes a list of cluster validity indexes, namely, Silhouette, Calinski-Harabasz, COP, and modified Davies-Bouldin index. The search space for window duration and overlap percentage were 120, 150, and 180 minutes and 0%, 25%, 50%, and 75%, respectively. The search space for window duration was chosen such that the duration is sufficient to capture the activity profile of rapid-acting insulin.

After determining the aforementioned hyperparameters, the number of patterns to be extracted by the DTW model has to be determined. A DTW model was trained for each of 3 to 8 patterns, and the models were compared. The optimal number of patterns was determined by evaluating the total within-cluster distance against the number of pattern graphs, which is also known as the elbow method. Finally, GV patterns and the type of pattern to which each series belongs were extracted from the best-performing DTW model. To examine whether our method can be generalized to other CGM data sets on patients with T1DM, we applied the same preprocessing steps and hyperparameters to the REPLACE-BG data set. The number of patterns was determined similarly, and the resulting set of GV patterns was compared with that from FSL data.

### Hierarchical Clustering of Patients and Statistical Analysis

Hierarchical clustering algorithm was used to cluster patients with respect to time in patterns, so that no a priori information about the number of clusters would be required [51]. The occurrence of each pattern per patient was tallied and expressed as a percentage of all patterns. Agglomerative hierarchical clustering algorithm with complete linkage was applied on time in patterns, and a dendrogram was plotted. A distance measure specific to percentage data was used for computing the distance matrix for hierarchical clustering instead of the conventional Euclidean distance measure [52]. The number of patient clusters was determined based on the greatest difference in the total within-cluster distance from the dendrogram. Each patient was assigned to one of the clusters for statistical analysis.

In statistical analysis, patient characteristics, including demographics, laboratory test results, diagnoses, and medications, were compared across patient clusters using univariate analysis. Laboratory test results for HbA_1c_ level and estimated glomerular filtration rate were categorized into groups and regarded as categorical variables in 2-tailed statistical tests. ANOVA for continuous variables and chi-square test for categorical or binary variables were performed, and the corresponding *P* values were extracted. Missing values for each variable were omitted from the computation of *P* value. *P* values <.05 were considered as being statistically significant.

### Ethics Approval

This study obtained ethics and data governance approval by the Royal Berkshire NHS Foundation Trust under the reference number A2901469.

## Results

### GV Patterns From DTW Model

A total of 1,590,443 CGM data points across 126 patients was collected in this study. After hyperparameter tuning, it was determined that 150 minutes was the optimal window duration and 50% was the optimal overlap percentage. A comparison of the cluster validity indexes is presented in Figure S1 in Multimedia Appendix 1. This resulted in 149,639 window periods (each 150-minute long) for training the DTW model. By evaluating the graph of the total within-cluster distance against the number of patterns, 6 was found to be the optimal number of patterns. In contrast, GV patterns from the REPLACE-BG data set were extracted with identical configurations, resulting in 931,005 window periods and 5 patterns (Figures S2 and S3 in Multimedia Appendix 1).

**Figure 2:**
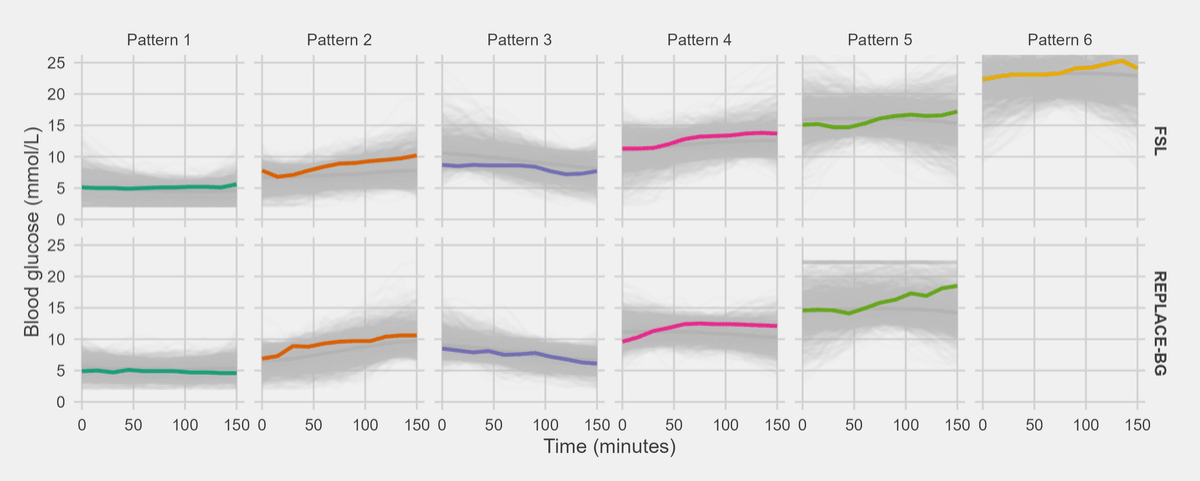
Glycemic variability patterns extracted from dynamic time warping model. Each gray line represents a random sample within the specific pattern and data set, and one is highlighted in color. The dark gray line in each panel depicts the median of glycemic variability patterns extracted. FSL: FreeStyle Libre.

The properties of the 6 GV patterns extracted from the FSL data set are summarized in Table 2. Figure 2 presents several random CGM samples from each pattern group. Results showed that patterns 1 and 2 represent glucose levels at approximately 3 to 6 mmol/L and 6 to 8 mmol/L, respectively, which mostly fall within the target range. A slightly rising trend is also observed in pattern 2. BG trends are also captured in patterns 3 and 4. Pattern 3 represents a decline in BG from marginally hyperglycemic to normal and is the only pattern that depicts an obvious downward trend. In contrast, pattern 4 represents a surge from marginally hyperglycemic to hyperglycemic. Most of the CGM data belong to patterns 1 to 4, and each of them accounts for approximately 20% of the data. Patterns 5 and 6 both represent less frequent hyperglycemic events at approximately 14 to 19 mmol/L and 19 to 28 mmol/L, respectively. Unlike the other 4 patterns, patterns 5 and 6 had large spread and included different trends that generally falls within their respective glucose levels. In other words, they can include upward, downward, steady, or even parabolic trends.

**Table 2:** Summary of the 6 glycemic variability (GV) patterns extracted from FreeStyle Libre data set.

GV pattern number	Glucose level	Pattern trends	Occurrence (N=149,639), n (%)
6	Severely hyperglycemic	Steady or rising to peak and declining	8440 (5.64)
5	Hyperglycemic	Steady or concave up or down	22,594 (15.10)
4	From marginally hyperglycemic to hyperglycemic	Rising	28,653 (19.15)
3	From marginally hyperglycemic to normal	Declining	31,185 (20.84)
2	Normal	Steady or slightly rising	30,255 (20.22)
1	Marginally hypoglycemic or normal	Steady or concave up	28,512 (19.05)

External validation was performed on the REPLACE-BG data set, and results are presented in Figure 2 and Figures S2 and S3 in Multimedia Appendix 1. It is observed that our methods were able to generate a comparable set of GV patterns across the 2 data sets, specifically, patterns 1 to 5. Compared with FSL patterns, the biggest difference in REPLACE-BG patterns is the absence of pattern 6, which indicates severe fluctuations in hyperglycemic events. This is likely owing to the difference in inclusion and exclusion criteria between the 2 data sets. The REPLACE-BG trial cohort deliberately included patients with T1DM who were well controlled and excluded individuals with substantial hypoglycemic events. Therefore, the REPLACE-BG data set is only representative of the well-controlled T1DM group and has limited generalizability to all patients with T1DM. Given that the objective of this study was to generate a comprehensive representation of GV profiles among all patients with T1DM, all further analysis in this study was conducted based on the 6 patterns from FSL data set.

### Patient GV Profile Clusters Based on Time in Patterns

**Figure 3:**
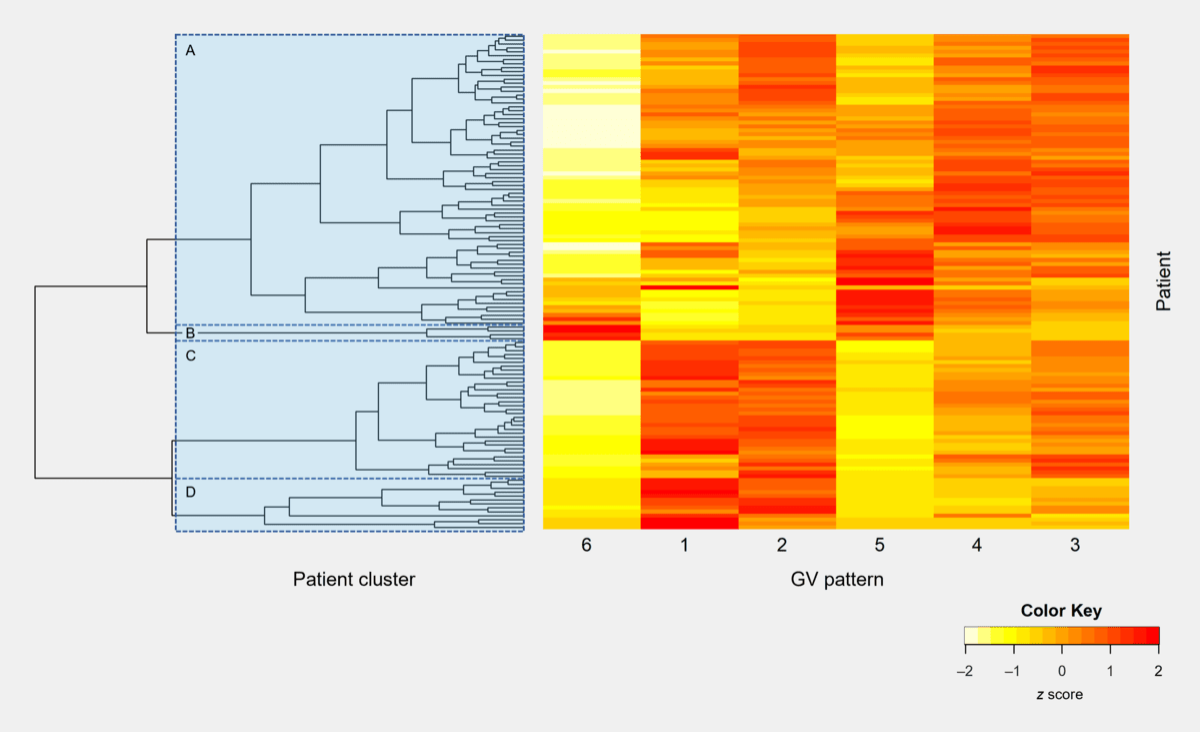
Dendrogram in hierarchical clustering and heat map of time in patterns per patient. The left panel depicts the dendrogram in hierarchical clustering. The 4 colored boxes represent 4 different patient clusters based on glycemic variability (GV) patterns. The right panel is a heat map that depicts the underlying distribution of patterns across all patients. Each row represents a patient and each column represents 1 of the 6 extracted GV patterns. Yellow color represents a relatively rare occurrence, and red color represents a relatively frequent occurrence.

Hierarchical clustering was applied on time in GV patterns. Overall, 4 clusters of patients were identified based on the dendrogram (Figure 3). Most patients (74/126, 58.7%) belonged to cluster A. Hyperglycemia fluctuation events occurred more frequently among patients in clusters A and B. Moreover, the time spent in GV patterns 1 and 2 for these patients was relatively low. In particular, the 3.2% (4/126) patients in cluster B spent much more time in GV pattern 6 than in all other clusters. This demonstrates that their glucose level was very poorly controlled and managed. In contrast, the glucose level of patients in clusters C and D are more likely to fall into GV patterns 1 and 2, which roughly resembles the target range. However, patients in cluster C spent relatively more time in GV patterns 3 and 4 when compared with patients in cluster D, which indicates great fluctuation in glucose levels and high likelihood of hyperglycemia events.

### Correlation Between GV-Based Clusters and Patient Characteristics

Patient characteristics were compared across the 4 patient clusters and are presented in Table 3. No statistical significance was found across clusters in terms of demographical variables, except for age (*P*=.02). Specifically, patients in cluster B were observed to be younger and had shorter duration of diabetes than those in the other 3 clusters (*P*=.04). Moreover, the patient clusters were significantly different in various glycemic metrics, including HbA_1c_ level category (*P*<.001), COV (*P*=.003), and TIR (*P*<.002). Patients in cluster D were associated with high odds of meeting the HbA_1c_ level and TIR recommended targets. Although more than half of patients in cluster C (23/35, 66%) met the recommended target for HbA_1c_ level, they had one of the greatest COV among all 4 clusters, and only 11% (4/35) of them met the recommended target for COV. Patients in clusters A and B were associated with significantly increased likelihood of poorly controlled diabetes. Most patients in cluster A and all patients in cluster B failed to fulfill HbA_1c_ level (7/74, 10%) and TIR targets, indicating further management needs in terms of type or dosage of insulin intake.

**Table 3:** Patient characteristics across the 4 patient clusters (N=126).

Characteristics				Cluster A (n=74)		Cluster B (n=4)		Cluster C (n=35)		Cluster D (n=13)		*P* value^a^	
Age (years), mean (SD)				40.3 (14.3)		22.8 (4.27)		41.8 (12.9)		33.8 (10.7)		*.02*	
Sex (female), n (%)				34 (46)		1 (25)		18 (51)		9 (69)		.33	
Index of Multiple Deprivation decile [53], mean (SD)				7.96 (2.31)		8.25 (2.06)		7.34 (2.44)		7.38 (2.66)		.56	
BMI (kg/m^2^), mean (SD)				27.3 (4.64)		24.1 (3.07)		26.9 (5.69)		23.4 (4.39)		.22	
Duration of diabetes (years), mean (SD)				22.2 (12.4)		8.5 (4.36)		24.8 (15.7)		14.5 (14.7)		*.04*	
Number of days since CGM^b^ use, mean (SD)				218 (243)		203 (56.1)		167 (203)		215 (276)		.76	
**eGFR^c^ stage, n (%)**													.85
	5			0 (0)		0 (0)		0 (0)		0 (0)			
	4			1 (1)		0 (0)		0 (0)		0 (0)			
	3b			2 (3)		0 (0)		0 (0)		0 (0)			
	3a			4 (5)		0 (0)		1 (3)		0 (0)			
	2			28 (38)		0 (0)		16 (46)		6 (46)			
	1			38 (51)		4 (100)		18 (51)		7 (54)			
**HbA_1c_^d^ level (mmol/mol), n (%)**													*<.001*
	≤42			1 (1)		0 (0)		1 (3)		7 (54)			
	43-48			4 (5)		0 (0)		6 (17)		3 (23)			
	48-59			14 (19)		0 (0)		19 (54)		2 (15)			
	59-85			46 (62)		1 (25)		9 (26)		1 (8)			
	≥86			8 (11)		3 (75)		0 (0)		0 (0)			
Glucose level, mean (SD)				10.8 (1.57)		19.3 (1.46)		8.22 (0.614)		6.48 (1.1)		*<.001*	
COV^e^ of glucose level, mean (SD)				0.428 (0.066)		0.354 (0.031)		0.429 (0.061)		0.37 (0.063)		*.003*	
**TIR^f^ (mmol/L), mean % (SD)**													
	≤3			1.9 (2.2)		0.3 (0.2)		3.2 (2.8)		4.6 (5)		*.002*	
	3-3.9			3.5 (2)		0.6 (0.2)		6.7 (2.7)		11 (7.7)		*<.001*	
	3.9-10			43.3 (11.3)		10.6 (2.9)		62.4 (6.4)		74.7 (12.9)		*<.001*	
	10-13.9			27.2 (6.0)		13.3 (3.6)		20.5 (4)		7.5 (4.9)		*<.001*	
	≥13.9			24.1 (11.6)		75.2 (5.8)		7.3 (3.4)		2.3 (5.8)		*<.001*	
**Time in patterns, mean % (SD)**													
	1			13.6 (7.2)		2.1 (0.6)		27.6 (8.8)		51.9 (17.9)		*<.001*	
	2			17 (6.1)		3.6 (1.1)		27.6 (5)		32.5 (10.6)		*<.001*	
	3			22.4 (4.7)		6.8 (1.7)		23.5 (5.2)		10.2 (6.6)		*<.001*	
	4			23.1 (5.7)		10.1 (2.8)		15.6 (4.2)		5 (7.3)		*<.001*	
	5			19.3 (7.8)		26.2 (5.8)		5.5 (3)		0.4 (0.7)		*<.001*	
	6			4.7 (6)		51.2 (11)		0.3 (0.4)		0 (0)		*<.001*	
**Fulfillment of recommended targets [28], n (%)**													
	TIR between 3.9 and 10 mmol/L >70% of the time			7 (10)		0 (0)		23 (66)		11 (85)		*<.001*	
	COV of glucose level <0.36			8 (11)		2 (50)		4 (11)		7 (54)		*<.001*	
	HbA_1c_ level <58 mmol/mol			18 (24)		0 (0)		26 (74)		12 (92)		*<.001*	
Comorbidities, n (%)				55 (74)		3 (75)		22 (63)		5 (39)		.10	
Diabetic complications, n (%)				47 (64)		3 (75)		19 (54)		4 (31)		.18	
**Medication, n (%)**													
	**Insulin**												
		Injection	66 (89)		4 (100)		33 (94)		12 (92)		.75		
		Pump	11 (15)		0 (0)		4 (11)		2 (15)		.78		
	Blood pressure			19 (26)		1 (25)		9 (26)		1 (8)		.55	
	Cholesterol			19 (26)		0 (0)		8 (23)		1 (8)		.33	
	Thyroid			10 (14)		0 (0)		2 (6)		2 (15)		.50	
	Antiplatelet			4 (5)		0 (0)		2 (6)		1 (8)		.95	
	Psychology			7 (10)		0 (0)		0 (0)		0 (0)		.15	

^a^*P* values <.05 are italicized; missing values were omitted only during the calculation of *P* values.

^b^CGM: continuous glucose monitoring.

^c^eGFR: estimated glomerular filtration rate.

^d^HbA_1c_: glycated hemoglobin.

^e^COV: coefficient of variation.

^f^TIR: time in range.

### Resemblance Between TIRs and Time in GV Patterns

**Figure 4:**
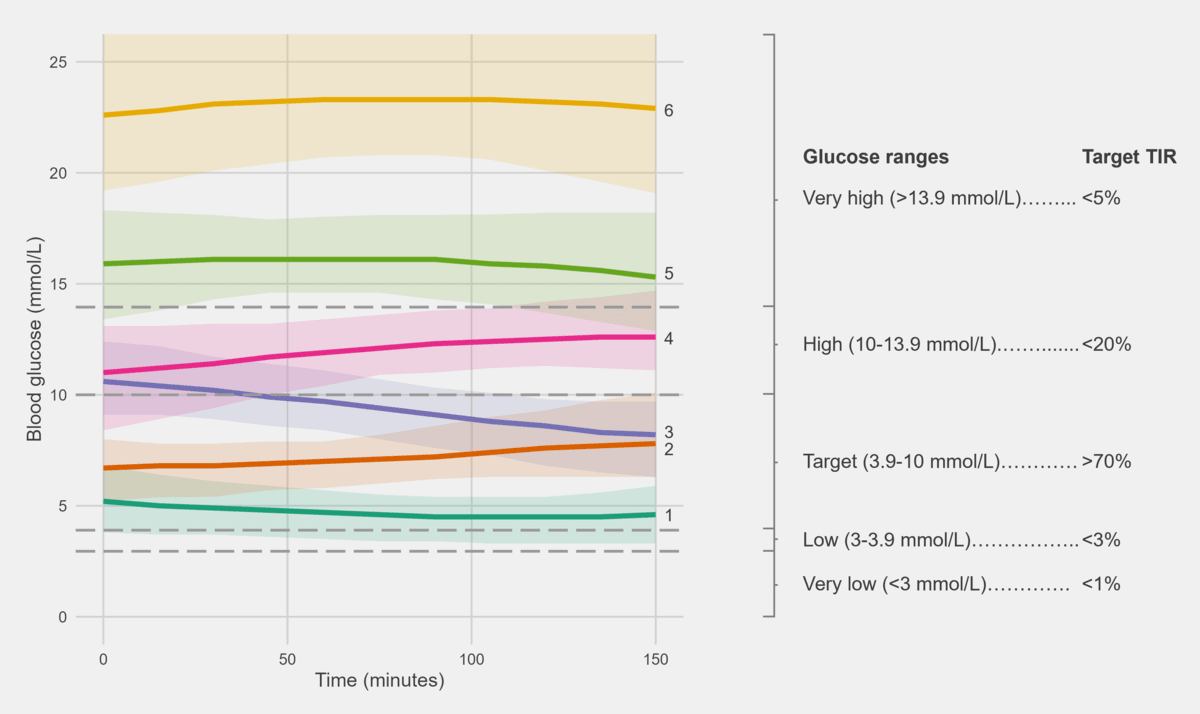
Comparison of recommended time in range (TIR) targets and extracted glycemic variability patterns. Each color in the left panel represents a glycemic variability pattern. The lower and upper bound of each shaded region represent the 20th and 80th percentile of glucose trend for that pattern. The median glucose trend of each pattern is highlighted. The target TIR shown in the right panel is proposed by Battelino et al [28].

It is possible to translate some of the TIR targets to targets of GV patterns owing to their conceptual similarity, and it is observed that some of the extracted GV patterns resemble the TIR glucose cutoff points as recommended by Battelino et al [28] (Figure 4). This can potentially serve as reference to better understand the clinical impacts for each pattern. GV patterns 5 and 6 both belong to the very high glucose range. Thus, a recommended target TIR of <5% within the very high glucose range can be approximately translated to having <5% occurrence for patterns 5 and 6. Pattern 4 generally represents high glucose level, with cutoffs at approximately 10 and 13.9 mmol/L. However, none of the patterns exclusively covers the very low glucose range (<3.9 mmol/L). This is because such readings were very rare in the data set, such that they were inherently grouped into GV pattern 1 by the DTW model.

As our extracted GV patterns take fluctuation in BG into account in addition to its magnitude, our method is able to provide additional context for a person’s BG profile. The prevalence of GV patterns 4 and 5 would indicate a fluctuation between high and very high glucose ranges, whereas that of GV patterns 3 and 4 indicates a fluctuation between target to high glucose level. This piece of information cannot be deduced from TIR. It should be noted that taking fluctuation into account also implies that direct translation from TIR targets to certain patterns is unavailable, as they span across different glucose ranges. For instance, the target glucose level ranges between 3.9 and 10 mmol/L comprises patterns 1, 2, and 3.

### GV Patterns Over Time

**Figure 5:**
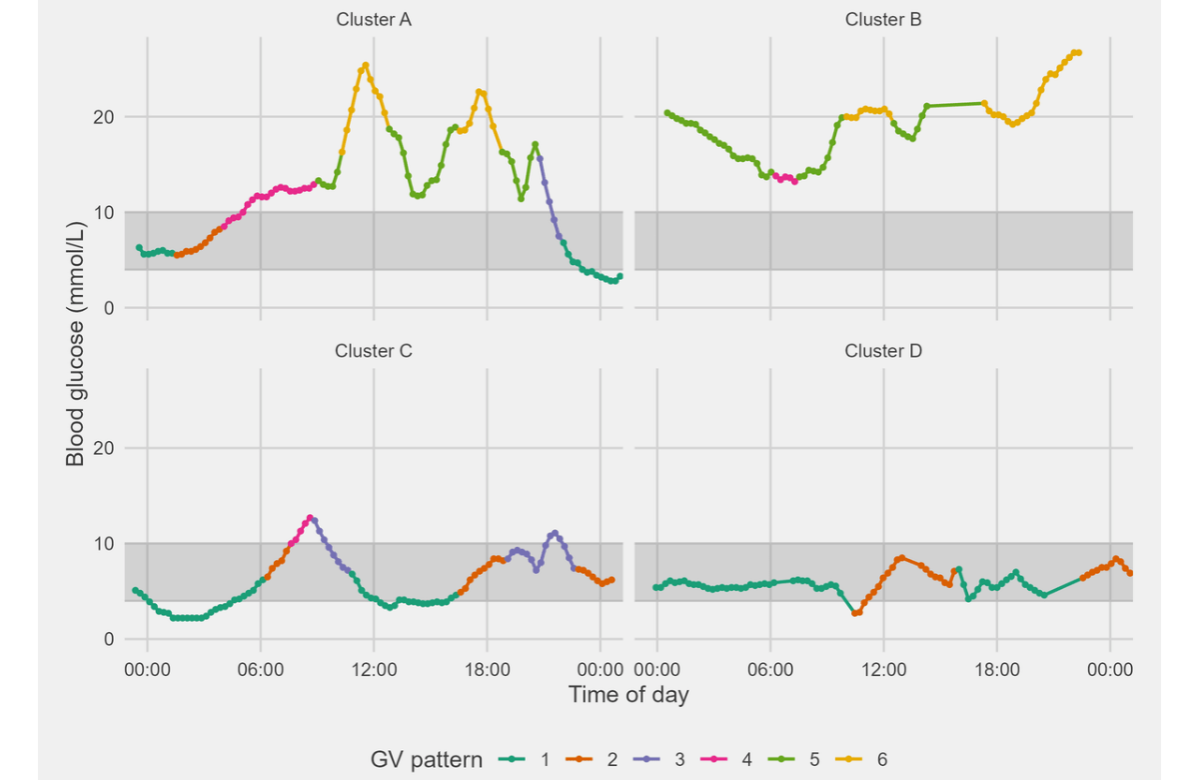
The 1-day glucose trend of patients sampled from each cluster. The shaded region represents the target glucose range, and the 6 glycemic variability (GV) patterns over time are highlighted in 6 colors.

In this study, we sought to draw insights about patients with different time in GV patterns by using hierarchical clustering. A total of 4 clusters was found, each with very distinguishing glycemic fluctuation features and thus management needs. An example of daily glucose trends from each cluster is presented in Figure 5. Diabetes in patients in cluster D was well controlled, and there is no need to alter their insulin regime. Although the glucose level of patients in cluster C usually falls within target range, it has great variability, which could indicate the need for changing their insulin regimes to reduce fluctuation and hyperglycemia events. In contrast, patients in clusters A and B had very poorly controlled diabetes, and a significant increase in fluctuation severity is observed, which suggests the need for change in glucose management. Patients in cluster A show sharp increases and decreases across target and hyperglycemia ranges, whereas those in cluster B primarily fluctuate at hyperglycemia level. A possible explanation for this is that patients in cluster B tend to be young and had short duration of diabetes. Therefore, the optimal way to manage their glucose levels is less apparent and would still require some time to be determined in follow-up consultations. Apart from existing metrics such as HbA_1c_ level and TIR, we believe that studying patient clusters can be beneficial as a complementary metric during consultations, which could improve patient care and, ultimately, clinical outcomes.

**Figure 6:**
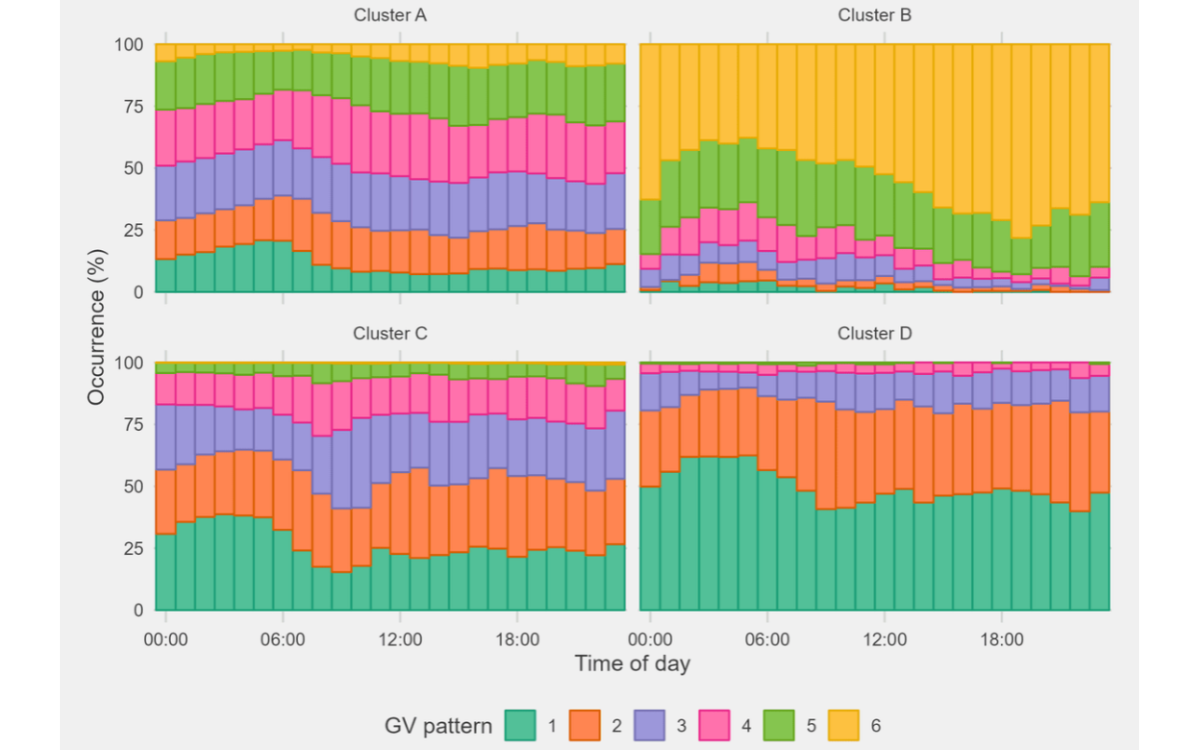
Hourly distribution of glycemic variability (GV) patterns across a day for each patient cluster.

To better understand the properties of each GV pattern, we further evaluated the relationship between GV patterns and time of day. The occurrence of patterns across time of day according to cluster is presented in Figure 6. It is observed that GV pattern 1, which represents steady glucose level around marginal hypoglycemia to normal, most frequently occurs at midnight between 2 AM and 6 AM. This is likely owing to the absence of food intake during the period. In contrast, GV patterns 2 and 4, which are indicators of a surge in glucose level, are more likely to occur at typical meal hours around 9 AM, 1 PM, and 7 PM for patients in clusters C and D. Similarly, GV patterns 5 and 6 occur the most within that period for patients in cluster B whose glucose level are very poorly controlled. These observations are generally consistent with existing literature about the daily fluctuation in glucose levels [29].

Apart from analyzing GV patterns over time of day, we further investigated whether the duration of CGM use is associated with patients’ GV profile and characteristics. On the basis of the distribution of CGM use duration in our data set, the cohort is divided into 3 approximately equal-sized groups to facilitate comparison: <68 days (46/126, 36.5%), 68 to 180 days (40/126, 31.7%), and >180 days (40/126, 31.7%). Our findings revealed that although no statistical significance was found between the duration of CGM use and patient demographics or fulfillment of recommended glycemic targets (all *P*>.05; Table 4), long duration is associated with specific glycemic metrics, including high mean glucose (*P*=.03), TIR ≥13.9 mmol/L (*P*=.04), and time in pattern 6 (*P*=.04). This may indicate increased likelihood of poorly controlled or managed patients who have been using CGM for an extended period.

**Table 4:** Patient characteristics across different duration of diabetes (N=126).

Characteristics			<68 days (n=46)		68-180 days (n=40)		>180 days (n=40)		*P* value^a^
Age (years), mean (SD)			38.7 (12.5)		40.4 (15.2)		39.4 (14)		.87
Sex (female), n (%)			26 (57)		16 (40)		20 (50)		.31
Index of Multiple Deprivation decile [53], mean (SD)			7.83 (2.01)		8.1 (2.34)		7.28 (2.73)		.28
BMI (kg/m^2^), mean (SD)			27.8 (6.2)		26.8 (3.9)		25.7 (4)		.22
Glucose level, mean (SD)			9.08 (1.57)		10.2 (2.5)		10.5 (3.5)		*.03*
COV^b^ of glucose level, mean (SD)			0.434 (0.074)		0.416 (0.06)		0.408 (0.061)		.17
**TIR^c^ (mmol/L), mean % (SD)**									
	≤3	3 (3.5)		1.7 (1.5)		2.6 (3)		.11	
	3-3.9	5.9 (3.6)		4.3 (2.8)		5 (5.3)		.19	
	3.9-10	55.3 (13.5)		49.1 (17.3)		47.3 (18.9)		.07	
	10-13.9	21.7 (7.2)		24.3 (8.5)		22.8 (9)		.36	
	≥13.9	14.1 (10.3)		20.6 (15.8)		22.4 (21.1)		*.04*	
**Time in patterns, mean % (SD)**									
	1	24.7 (14.5)		17.4 (13.5)		20.5 (17.5)		.09	
	2	23.4 (8.8)		20.2 (8.8)		19.4 (9.7)		.10	
	3	21.2 (7)		21.7 (6.3)		19.9 (7.1)		.46	
	4	17.8 (7.8)		20.1 (8.2)		18.3 (8.3)		.40	
	5	11.1 (9.2)		15.6 (9.4)		14.8 (11.1)		.08	
	6	1.7 (2.7)		4.9 (8.7)		7.1 (14.9)		*.04*	
**Fulfillment of recommended targets [28], n (%)**									
	TIR between 3.9 and 10 mmol/L >70% of the time	17 (37)		11 (28)		13 (33)		.65	
	COV of glucose level <0.36	7 (15)		8 (20)		6 (15)		.79	
	HbA_1c_^d^ level <58 mmol/mol	19 (41)		17 (43)		20 (50)		.61	

^a^*P* values <.05 are italicized; missing values were omitted only during the calculation of *P* values.

^b^COV: coefficient of variation.

^c^TIR: time in range.

^d^HbA_1c_: glycated hemoglobin.

## Discussion

### Principal Findings

As an important application of smart and connected health, CGM has been gaining popularity rapidly ever since its inception and is becoming a vital tool to improve glucose management in patients with T1DM. With the increasing use of CGM for managing patients with T1DM, metrics such as TIR are recommended to depict GV, but a significant part of information available in CGM data is often omitted. In this study, we proposed a machine learning framework for extracting GV patterns from CGM data that harnesses the strengths of machine learning in terms of the capability of analyzing large amounts of data. By applying DTW on CGM data, we showed that it is possible to extract recurring patterns in CGM that inherit the clinical concepts of TIR, a recognized CGM-derived metric. Specifically, 6 distinctive patterns were found, and we showed that time in patterns can be used to comprehensively represent patients’ GV profile and to complement TIR owing to their conceptual resemblance. We further drew insights from GV patterns by identifying the types of patients with T1DM based on time in patterns and addressing the relationship between GV patterns and time of day. Our method captured information beyond absolute glucose value and revealed the details of glucose variability and dynamics. We demonstrated that time in patterns is an accessible, more comprehensive representation of a patient’s GV and could provide additional insights such as types of patients with T1DM and time of day.

Our proposed methods successfully captured GV patterns that inherently incorporate the idea of clinically meaningful concepts such as mean glucose level, GV, and TIR. Time in patterns derived from our methods contains much rich information, as existing methods such as TIR disregard the sequence in which the glucose measurements were made. Finally, an advantage of our time-in-patterns method over other proposed machine learning–based metrics is its scalability and understandability, which is largely owing to the ability to visualize our extracted patterns from blood monitoring data. As mentioned in section *Quantifying GV*, clinical understandability is a major issue that hindered machine learning–based GV extraction methods from being a widely accepted glycemic metric. For example, it is generally more meaningful to portray GV using time in patterns, such as 36% time spent in GV pattern 3 (rising from marginally hyperglycemic to normal) and pattern 4 (declining from marginally hyperglycemic to hyperglycemic), than a single SD value such as 0.36. We also validated the blood fluctuation patterns 1 to 5 using US-based CGM data from the REPLACE-BG trial of 225 adults with well-controlled T1DM. This shows that our method has the generalizability to cover different patient cohorts from various demographics.

### Limitations

This study had a few limitations. First, the duration of CGM use varied from 1 month to 3 years across patients in this study. Although no significant association was found between days since the use of CGM and patient cluster (*P*=.76), certain effects may not be accounted for in this study, such as seasonal effects on glucose levels [54]. Second, the adoption of CGM at the moment is still limited to the well-developed areas of the world where there are information and communication technology infrastructure with high level of digital readiness for connected health and sufficient funding for patients with T1DM to use wearable CGM devices. This is also reflected in our data that the patients included in this study were predominantly living in less deprived areas. For example, 75.4% (95/126) of the patients in our study were living in less deprived areas according to the Index of Multiple Deprivation (IMD) decile (IMD≥7), and 34.9% (44/126) of them were living in the least deprived area (IMD=10). Only 19.8% (25/126) of the patients in our study were living in more deprived areas (IMD≤5). The average IMD decile in different patient clusters can be found in Table 3. Therefore, the generalizability of our results to other demographics such as patients living in rural areas is limited. It should also be noted that apart from infrastructure and deprivation, there are other factors affecting the adoption of CGM such as device accuracy [55], user perception, device obtrusiveness [56], and interpersonal influence [57]. Third, as only the latest list of medication and laboratory test results was collected from each patient, any change in medication or management throughout the study period was not accounted for. A patient who spent a lot of time in hyperglycemia may remain in the target glucose range steadily after a change in their insulin regime. In this case, the resulting time in patterns would be averaged across the 2 states and fail to represent the patient’s latest situation.

### Future Studies

Future studies could focus on investigating the clinical relationship between GV patterns and DM medications through prospective studies and randomized control trials. By having a more comprehensive representation of GV profile, we can better categorize patients, which in turn would enable us to understand more about their unique condition and needs. We believe that this framework can ultimately serve as a step toward the development of personalized therapeutic pathways for patients with DM in the environment of connected health.

## Appendix

Multimedia Appendix 1 Supplemental tables and figures.

## Abbreviations

BG: blood glucose
CGM: continuous glucose monitoring
COV: coefficient of variation
DM: diabetes mellitus
DTW: dynamic time warping
EPR: electronic patient record
FSL: FreeStyle Libre
GV: glycemic variability
HbA_1c_: glycated hemoglobin
IMD: Index of Multiple Deprivation
NHS: National Health Service
T1DM: type 1 diabetes mellitus
TIR: time in range

## Data Availability

The anonymized data set of this study was collected from the Centre for Diabetes and Endocrinology at the Royal Berkshire National Health Service Foundation Trust, the United Kingdom. The data set may be available subject to ethics and information governance approval from Royal Berkshire National Health Service Foundation Trust. The validation data set is from the REPLACE-BG randomized trial conducted in the United States among adults with well-controlled type 1 diabetes [47].

## References

[1] (2021). IDF Diabetes Atlas 10th edition. International Diabetes Federation.

[2] (2020). Global health estimates: Leading causes of death. Cause-specific mortality, 2000–2019. World Health Organization.

[3] Tamborlane WV, Beck RW, Bode BW, Buckingham B, Chase HP, Clemons R, Fiallo-Scharer R, Fox LA, Gilliam LK, Hirsch IB, Huang ES, Kollman C, Kowalski AJ, Laffel L, Lawrence JM, Lee J, Mauras N, O'Grady M, Ruedy KJ, Tansey M, Tsalikian E, Weinzimer S, Wilson DM, Wolpert H, Wysocki T, Xing D, Juvenile Diabetes Research Foundation Continuous Glucose Monitoring Study Group (2008). Continuous glucose monitoring and intensive treatment of type 1 diabetes. N Engl J Med.

[4] Reddy N, Verma N, Dungan K (2020). Monitoring technologies- continuous glucose monitoring, mobile technology, biomarkers of glycemic control. Endotext.

[5] Foster NC, Beck RW, Miller KM, Clements MA, Rickels MR, DiMeglio LA, Maahs DM, Tamborlane WV, Bergenstal R, Smith E, Olson BA, Garg SK (2019). State of type 1 diabetes management and outcomes from the T1D exchange in 2016-2018. Diabetes Technol Ther.

[6] Janapala RN, Jayaraj JS, Fathima N, Kashif T, Usman N, Dasari A, Jahan N, Sachmechi I (2019). Continuous glucose monitoring versus self-monitoring of blood glucose in type 2 diabetes mellitus: a systematic review with meta-analysis. Cureus.

[7] Huisman TH, Martis EA, Dozy A (1958). Chromatography of hemoglobin types on carboxymethylcellulose. J Lab Clin Med.

[8] Bookchin RM, Gallop PM (1968). Structure of hemoglobin AIc: nature of the N-terminal beta chain blocking group. Biochem Biophys Res Commun.

[9] Nathan DM, Turgeon H, Regan S (2007). Relationship between glycated haemoglobin levels and mean glucose levels over time. Diabetologia.

[10] Dhatariya K, Levy N, Kilvert A, Watson B, Cousins D, Flanagan D, Hilton L, Jairam C, Leyden K, Lipp A, Lobo D, Sinclair-Hammersley M, Rayman G, Joint British Diabetes Societies (2012). NHS diabetes guideline for the perioperative management of the adult patient with diabetes. Diabet Med.

[11] American Diabetes Association (2019). Standards of medical care in diabetes-2019 abridged for primary care providers. Clin Diabetes.

[12] Wilmot EG, Lumb A, Hammond P, Murphy HR, Scott E, Gibb FW, Platts J, Choudhary P (2021). Time in range: a best practice guide for UK diabetes healthcare professionals in the context of the COVID-19 global pandemic. Diabet Med.

[13] Suh S, Kim JH (2015). Glycemic variability: how do we measure it and why is it important?. Diabetes Metab J.

[14] Beck RW, Connor CG, Mullen DM, Wesley DM, Bergenstal RM (2017). The fallacy of average: how using HbA1c alone to assess glycemic control can be misleading. Diabetes Care.

[15] Cardoso CR, Leite NC, Moram CB, Salles GF (2018). Long-term visit-to-visit glycemic variability as predictor of micro- and macrovascular complications in patients with type 2 diabetes: the Rio de Janeiro Type 2 Diabetes Cohort Study. Cardiovasc Diabetol.

[16] Hirsch IB, Brownlee M (2005). Should minimal blood glucose variability become the gold standard of glycemic control?. J Diabetes Complications.

[17] Bellaver P, Schaeffer AF, Dullius DP, Viana MV, Leitão CB, Rech TH (2019). Association of multiple glycemic parameters at intensive care unit admission with mortality and clinical outcomes in critically ill patients. Sci Rep.

[18] Todi S, Bhattacharya M (2014). Glycemic variability and outcome in critically ill. Indian J Crit Care Med.

[19] Krinsley JS (2008). Glycemic variability: a strong independent predictor of mortality in critically ill patients. Crit Care Med.

[20] Oh TK, Heo E, Song IA, Jeong WJ, Han M, Bang JS (2020). Increased glucose variability during long-term therapeutic hypothermia as a predictor of poor neurological outcomes and mortality: a retrospective study. Ther Hypothermia Temp Manag.

[21] Gorst C, Kwok CS, Aslam S, Buchan I, Kontopantelis E, Myint PK, Heatlie G, Loke Y, Rutter MK, Mamas MA (2015). Long-term glycemic variability and risk of adverse outcomes: a systematic review and meta-analysis. Diabetes Care.

[22] Service FJ (2013). Glucose variability. Diabetes.

[23] Siegelaar SE, Holleman F, Hoekstra JB, DeVries JH (2010). Glucose variability; does it matter?. Endocr Rev.

[24] Omar AS, Salama A, Allam M, Elgohary Y, Mohammed S, Tuli AK, Singh R (2015). Association of time in blood glucose range with outcomes following cardiac surgery. BMC Anesthesiol.

[25] Beck RW, Bergenstal RM, Riddlesworth TD, Kollman C, Li Z, Brown AS, Close KL (2019). Validation of time in range as an outcome measure for diabetes clinical trials. Diabetes Care.

[26] Lu J, Ma X, Zhou J, Zhang L, Mo Y, Ying L, Lu W, Zhu W, Bao Y, Vigersky RA, Jia W (2018). Association of time in range, as assessed by continuous glucose monitoring, with diabetic retinopathy in type 2 diabetes. Diabetes Care.

[27] Beyond A1C Writing Group (2018). Need for regulatory change to incorporate beyond A1C glycemic metrics. Diabetes Care.

[28] Battelino T, Danne T, Bergenstal RM, Amiel SA, Beck R, Biester T, Bosi E, Buckingham BA, Cefalu WT, Close KL, Cobelli C, Dassau E, DeVries JH, Donaghue KC, Dovc K, Doyle 3rd FJ, Garg S, Grunberger G, Heller S, Heinemann L, Hirsch IB, Hovorka R, Jia W, Kordonouri O, Kovatchev B, Kowalski A, Laffel L, Levine B, Mayorov A, Mathieu C, Murphy HR, Nimri R, Nørgaard K, Parkin CG, Renard E, Rodbard D, Saboo B, Schatz D, Stoner K, Urakami T, Weinzimer SA, Phillip M (2019). Clinical targets for continuous glucose monitoring data interpretation: recommendations from the international consensus on time in range. Diabetes Care.

[29] Advani A (2020). Positioning time in range in diabetes management. Diabetologia.

[30] Murphy HR (2019). Continuous glucose monitoring targets in type 1 diabetes pregnancy: every 5% time in range matters. Diabetologia.

[31] Struble N (2013). Measuring Glycemic Variability and Predicting Blood Glucose Levels: Using Machine Learning Regression Models.

[32] Marling CR, Struble NW, Bunescu RC, Shubrook JH, Schwartz FL (2013). A consensus perceived glycemic variability metric. J Diabetes Sci Technol.

[33] Eljil KS, Qadah GZ, Pasquier M (2016). Predicting hypoglycemia in diabetic patients using time-sensitive artificial neural networks. Int J Healthc Inf Syst Inform.

[34] Mani S, Chen Y, Elasy T, Clayton W, Denny J (2012). Type 2 diabetes risk forecasting from EMR data using machine learning. AMIA Annu Symp Proc.

[35] Hall H, Perelman D, Breschi A, Limcaoco P, Kellogg R, McLaughlin T, Snyder M (2018). Glucotypes reveal new patterns of glucose dysregulation. PLoS Biol.

[36] Umpierrez GE, P Kovatchev B (2018). Glycemic variability: how to measure and its clinical implication for type 2 diabetes. Am J Med Sci.

[37] Rodbard D (2011). Clinical interpretation of indices of quality of glycemic control and glycemic variability. Postgrad Med.

[38] Rama Chandran S, Tay WL, Lye WK, Lim LL, Ratnasingam J, Tan AT, Gardner DS (2018). Beyond HbA1c: comparing glycemic variability and glycemic indices in predicting hypoglycemia in type 1 and type 2 diabetes. Diabetes Technol Ther.

[39] McDonnell CM, Donath SM, Vidmar SI, Werther GA, Cameron FJ (2005). A novel approach to continuous glucose analysis utilizing glycemic variation. Diabetes Technol Ther.

[40] Service FJ, Molnar GD, Rosevear JW, Ackerman E, Gatewood LC, Taylor WF (1970). Mean amplitude of glycemic excursions, a measure of diabetic instability. Diabetes.

[41] Kovatchev BP, Cox DJ, Gonder-Frederick LA, Clarke W (1997). Symmetrization of the blood glucose measurement scale and its applications. Diabetes Care.

[42] Hill NR, Hindmarsh PC, Stevens RJ, Stratton IM, Levy JC, Matthews DR (2007). A method for assessing quality of control from glucose profiles. Diabet Med.

[43] Zhu T, Uduku C, Li K, Herrero P, Oliver N, Georgiou P (2022). Enhancing self-management in type 1 diabetes with wearables and deep learning. NPJ Digit Med.

[44] R Core Team (2019). R: a language and environment for statistical computing. R Foundation for Statistical Computing.

[45] Sarda-Espinosa A (2023). dtwclust: time series clustering along with optimizations for the dynamic time warping distance. The Comprehensive R Archive Network.

[46] (2017). FreeStyle Libre for glucose monitoring. National Institute for Health and Care Excellence.

[47] Aleppo G, Ruedy KJ, Riddlesworth TD, Kruger DF, Peters AL, Hirsch I, Bergenstal RM, Toschi E, Ahmann AJ, Shah VN, Rickels MR, Bode BW, Philis-Tsimikas A, Pop-Busui R, Rodriguez H, Eyth E, Bhargava A, Kollman C, Beck RW, REPLACE-BG Study Group (2017). REPLACE-BG: a randomized trial comparing continuous glucose monitoring with and without routine blood glucose monitoring in adults with well-controlled type 1 diabetes. Diabetes Care.

[48] Berndt DJ, Clifford J (1994). Using dynamic time warping to find patterns in time series. Proceedings of the 3rd International Conference on Knowledge Discovery and Data Mining.

[49] Mazandarani FN, Mohebbi M (2018). Wide complex tachycardia discrimination using dynamic time warping of ECG beats. Comput Methods Programs Biomed.

[50] Skutkova H, Vitek M, Babula P, Kizek R, Provaznik I (2013). Classification of genomic signals using dynamic time warping. BMC Bioinformatics.

[51] Govender P, Sivakumar V (2020). Application of k-means and hierarchical clustering techniques for analysis of air pollution: a review (1980–2019). Atmos Pollut Res.

[52] Edwards AW (1971). Distances between populations on the basis of gene frequencies. Biometrics.

[53] (2019). English indices of deprivation 2019. National Statistics, UK.

[54] Jarrett RJ, Murrells TJ, Shipley MJ, Hall T (1984). Screening blood glucose values: effects of season and time of day. Diabetologia.

[55] Cappon G, Vettoretti M, Sparacino G, Facchinetti A (2019). Continuous glucose monitoring sensors for diabetes management: a review of technologies and applications. Diabetes Metab J.

[56] Engler R, Routh TL, Lucisano JY (2018). Adoption barriers for continuous glucose monitoring and their potential reduction with a fully implanted system: results from patient preference surveys. Clin Diabetes.

[57] Hossain MI, Yusof AF, Hussin AR, lahad NA, Sadiq AS (2021). Factors influencing adoption model of continuous glucose monitoring devices for internet of things healthcare. Internet Things.

