# Supplementary material for: Machine Learning–Based Time in Patterns for Blood Glucose Fluctuation Pattern Recognition in Type 1 Diabetes Management: Development and Validation Study
Source: JMIR AI. 2023 May 26;2:e45450. doi: 10.2196/45450 (PMC11041419; doi:10.2196/45450)
Supplement: Multimedia Appendix 1 [file ai_v2i1e45450_app1.pdf]

This is a Multimedia Appendix to a full manuscript published in the J Med Internet Res. For full copyright and citation information see http://dx.doi.org/10.2196/45450.

Table S1. Categorization of medications.

Category	Type of Insulin drug	Medication
Anitbiotics		Lymecycline
Antihistamin		Cetirizine
Blood Pressure		Amlodipine
		Candesartan
		Diltiazem MR
		Hydralazine
		Indapamide
		Lercanidipine
		Losartan
		Losartan Prava
		Perindopril
		Ramipril
Antiplatelet		Aspirin
		Clopidogrel
Cholesterol		Atorvastatin
		Pravastatin
		Simvastatin
		Statin
DM	Injection (long-acting)	Degludec
		Glargine
		Lantus
		Levemir
		Tresiba
		Toujeo
	Injection (short-acting)	Apidra
		Fiasp
		Humalog
		NovoRapid
		Humulin
	Pump	CSII
		Humalog CSII
		NovoRapid CSII
Immunosuppression		Azathioprine

Category	Type of Insulin drug	Medication
Kidney test		Creatine
Neuro		Duloxetine
Psychology		Carbamazepine
		Cerelle
		Citalopram
Thyroid	Levothyroxine	
		Thyroxine

Table S2. Categorization of diagnosis.

Category	Diagnosis
Diabetic complication	Angioplasty (foot)
	Foot amputation
	Maculopathy
	Nephropathy
	Neuropathy
	Retinopathy
	Inactive Charcot foot
	Sensory neuropathy
Metabolic comorbidities	Angioplasty (cardiac)
	Dyslipidemia
	Hypertension
	Peripheral vascular disease
	Posterior circulation stroke
	TIA
Psychology	Anxiety
	Depression

Figure S1. Comparison of cluster validity indices.

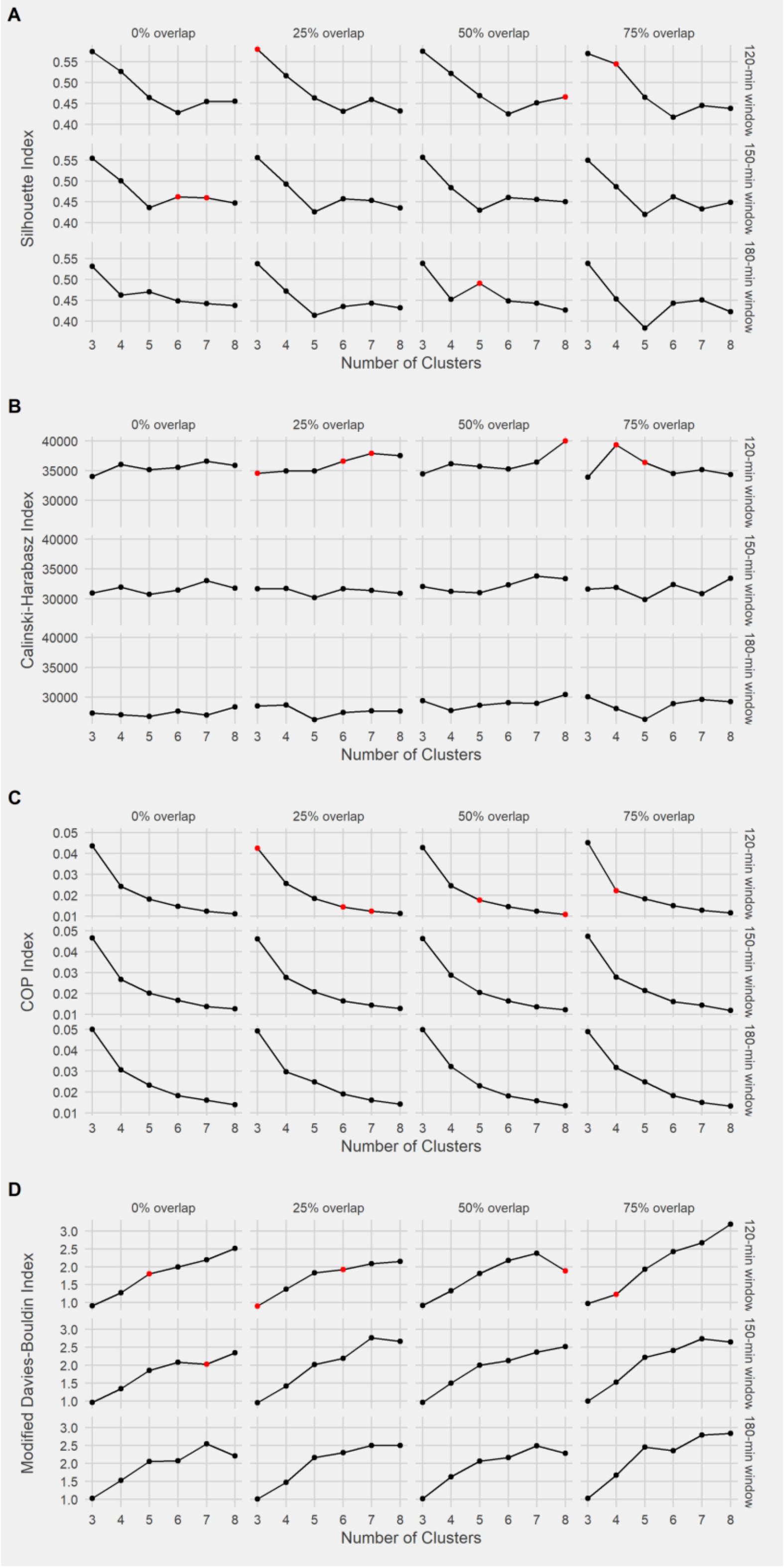

Red dots represent the best performing model among the search space of overlap percentage and window duration.

Figure S2. Total within-cluster distance against number of patterns.

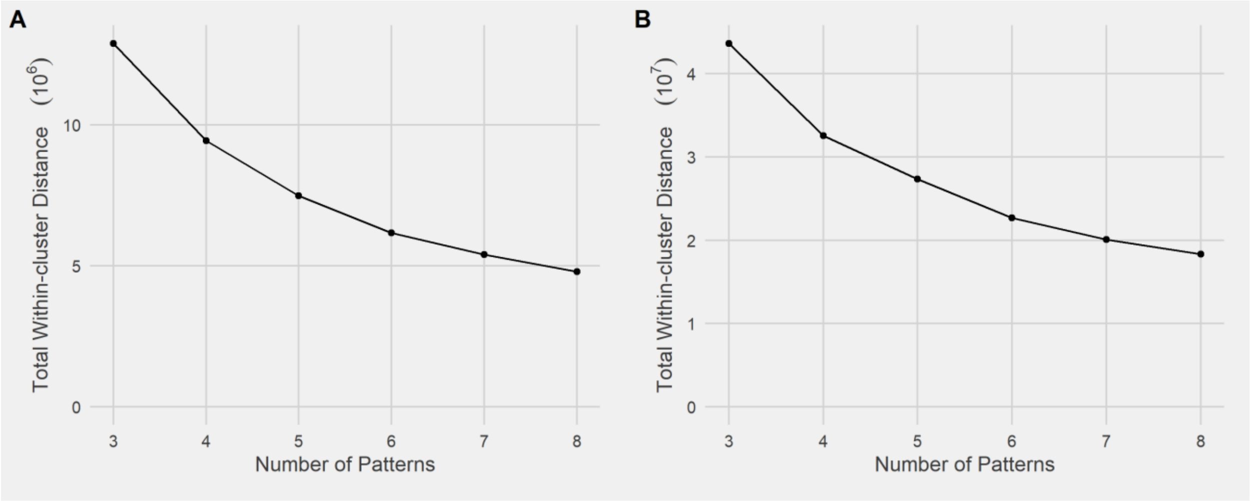

(A) FSL dataset. (B) REPLACE-BG dataset.

Figure S3. Comparison of the median value of GV patterns extracted with different number of clusters

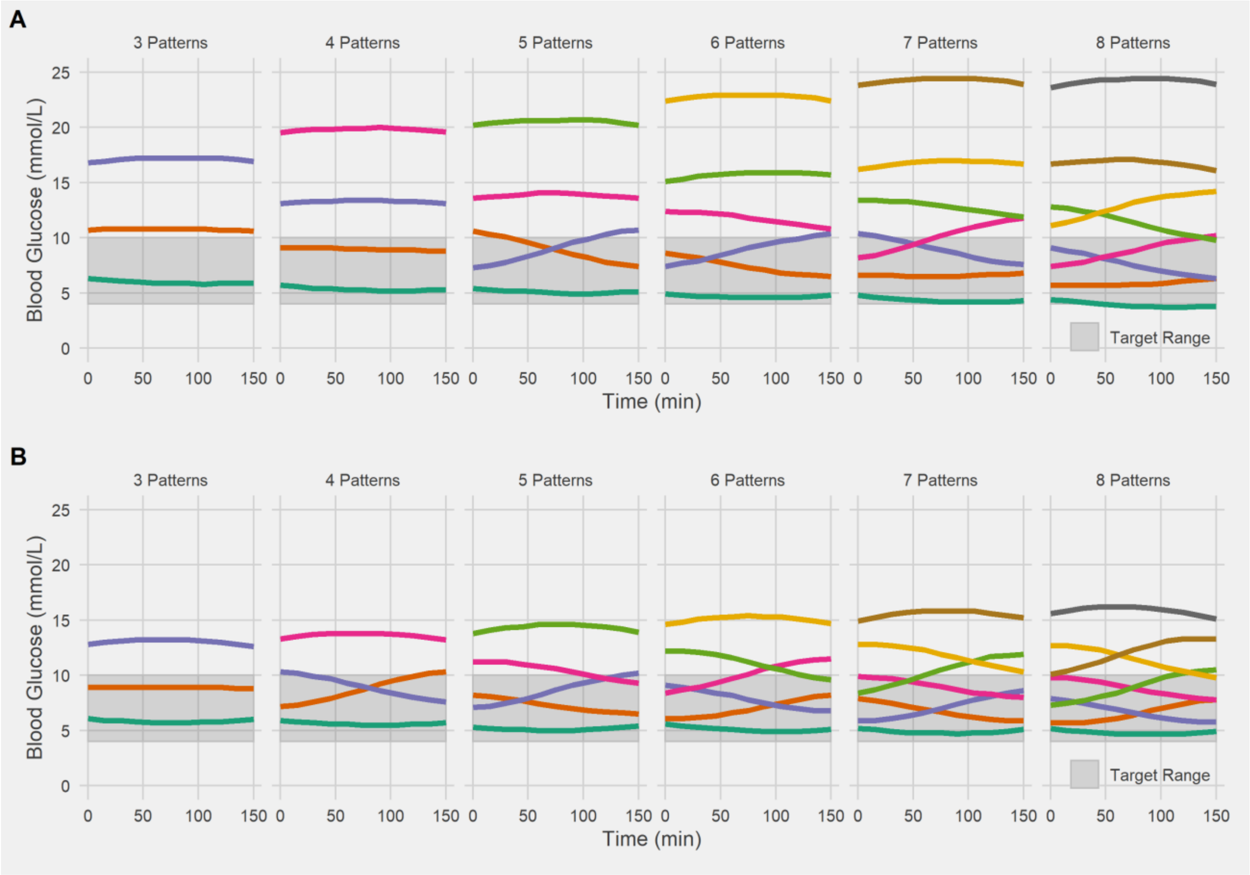

(A) FSL dataset. (B) REPLACE-BG dataset.
